# The Impacts of Uncertainty Stress on Mental Disorders of Chinese College Students: Evidence From a Nationwide Study

**DOI:** 10.3389/fpsyg.2020.00243

**Published:** 2020-03-10

**Authors:** Dan Wu, Lingwei Yu, Tingzhong Yang, Randall Cottrell, Sihui Peng, Wei Guo, Shuhan Jiang

**Affiliations:** ^1^Department of Psychology/Research Center on Quality of Life and Applied Psychology, Guangdong Medical University, Dongguan, China; ^2^Department of Social Medicine/Center for Tobacco Control Research, School of Medicine, Zhejiang University, Hangzhou, China; ^3^Children’s Hospital, Zhejiang University School of Medicine, Hangzhou, China; ^4^Public Health Studies Program, University of North Carolina Wilmington, Wilmington, NC, United States; ^5^Hangzhou Hospital for the Prevention and Treatment of Occupational Diseases, Hangzhou, China; ^6^School of Humanities and Management, Zhejiang Medical University, Hangzhou, China

**Keywords:** uncertainty stress, study stress, life stress, mental disorders, Chinese college students

## Abstract

**Objectives:** This study aimed to examine the relationships between the types of stress and students’ mental health, to distinguish the effects of stressors on mental health problems, and to explore the important role of uncertainty stress on the development of mental disorders in a nationally representative sample of Chinese college students.

**Methods:** A cross-sectional multistage study was conducted. Participants were 11,954 students, who were recruited from 50 Chinese universities located in 43 cities covering 23 provinces, autonomous regions, and municipalities across China. The Student Daily Stress Questionnaire (SDSQ) was applied to measure the different types of stress, and mental health status was measured using the 12-item Chinese Health Questionnaire (CHQ). Both unadjusted and adjusted logistic regression models were utilized in the statistical analyses. Multilevel analyses were performed to examine the variation of mental disorder at both the individual and university levels.

**Results:** The prevalence of mental disorders was 22.8% (95% CI: 22.0–23.5%). The unadjusted models showed that age, gender, grade, major, and university location and type were the correlates of mental disorders among students. The unadjusted models developed in this research found that study stress, life stress, and uncertainty stress were positively associated with mental disorder. The multilevel logistic regression models showed that uncertainty stress was far more likely to result in students’ mental disorders than study or life stress after controlling for university level. The greater the perceived uncertainty stress, the higher the prevalence of mental disorders.

**Conclusion:** This study provides robust evidence of the impact of uncertainty stress on mental disorders among college students. Compared with life and study stress, more attention should be given to uncertainty stress. The information from this study should be helpful when considering effective mental health policies and interventions among college students in China.

## Introduction

The issue of mental health among college students is of increasing concern (Karatekin, 2018). The college years are a peak period for the onset of mental disorder in which young people experience a unique stage of psycho-social development and transition from late adolescence to emerging adulthood (Wu et al., 2016; Cuijpers et al., 2019). College students are an important population segment in determining the economic growth and success of a country (Auerbach et al., 2018). The adverse effects of mental disorders during this period are profound including dropping out of college, poor academic performance, strained relationships, and reduced emotional functioning. Together, these effects can have a negative impact on physical health and future professional career potential (Cuijpers et al., 2019). Mental health problem in this population should be a key public health priority. However, most research on mental disorders among students has focused on the primary and secondary school years (Auerbach et al., 2016). Not enough attention has been paid to identify mental disorders among college students (Auerbach et al., 2018).

It is well known that college students experience high levels of stress (Holland, 2016). There is strong evidence that the cumulative impact of stress has been associated with mental health problems, which are prevalent in the college population (Lee et al., 2004; American College Health Association, 2009; Holland, 2016). The general strain theory argues that individuals who are stressed are more likely to experience negative affective states such as anger, fear, and frustration. These affective states, in turn, create an internal pressure, which can lead to the strain outcomes (Agnew, 1992). The social stress theory also points out that stressful life events play an important role in producing emotional and behavior outcomes for individuals, which could raise the risk of mental disorder (Aneshensel, 1992). Mental health problems can cause life stress. On the other hand, the causal link can also operate in the opposite direction, with life stress leading to mental health problems (Aneshensel, 1992; Silver and Teasdale, 2005). The more the occurrence of negative events, the greater the likelihood that a disorder will develop (Dohrenwend, 2000). The relationship between stress and poor mental health has been demonstrated in both prospective and retrospective studies from multiple countries (Kitzrow, 2003; Lee et al., 2004; Karatekin, 2018).

Study stress and general life stress are very common types of stress among college students (Misra and McKean, 2000; Holland, 2016). Study stress is specific to students and measures the amount of stress students experience related to such events as curriculum changes, exams, papers, grade competition, and so forth (Misra and McKean, 2000). Life stress focuses more on the major life events that impact an individual and the resulting stress from these life events, such as a poor living situation, health conditions, interpersonal relationships, and others (Yang et al., 2017b). There is accumulating evidence that life stress and study stress are salient risk factors that contribute to college students’ mental health (Kitzrow, 2003; Andrews and Wilding, 2004; Lee et al., 2004). Both life stress and study stress tend to be fairly predictable and are considered to be certainty stressors. In contrast to certainty stress, uncertainty stress is persistent experiences that require more psychological readjustments (Peng et al., 2019). However, the mechanism between uncertainty stress and mental disorder was still unknown.

In the past decades, researchers have paid increased attention to uncertainty (Mishel, 1988; Greco and Roger, 2003; McEvoy and Mahoney, 2011; Peters et al., 2017). Uncertainty was defined as “incomplete information or knowledge about a situation, or the possible alternatives or the probability of their occurrence, their outcomes are not known by the subjects” in accordance with the stress cognition theory (Scholz, 1983). Uncertainty is a major cognitive and psychological stressor (Greco and Roger, 2003). Individuals who are intolerant of uncertainty tend to perceive it as stressful and then respond negatively on emotional, cognitive, and behavioral levels (McEvoy and Mahoney, 2011). Uncertainty constitutes a stressful condition that arouses a stress response that then contributes to ill health (Monat et al., 1972; Peters et al., 2017). The literature on uncertainty initially focused on clinical settings (Mishel, 1988; Carleton et al., 2012). There is a large number of studies from countries outside China that demonstrate that uncertainty plays a significant role in explaining various illness outcomes, especially in cancer patients, psychiatric patients, and chronic disease patients (Kang, 2005; Sammarco and Konecny, 2008; Jiang and He, 2012). Uncertainty has a negative impact on quality of life in breast cancer survivors and is related to health and functioning, socioeconomic status, and psychological and spiritual well being (Sammarco and Konecny, 2008). The phenomenon of uncertainty has also been associated with a range of psychological maladies including depression, anxiety, and psychiatric symptoms among children with chronic illness (Stewart and Mishel, 2000). Evidence is accumulating that intolerance of uncertainty may be a transdiagnostic maintaining factor across anxiety disorders and depression (McEvoy and Mahoney, 2011). Uncertainty has also been found to explain the unique variance with neuroticism as well as with symptoms related to various anxiety disorders and depression (McEvoy and Mahoney, 2012).

Despite the growing literature relating uncertainty to increased psychological distress in the context of illness and hospitalization, it remains unclear how this association and mechanism impacts the general population (Carleton et al., 2012). To address this research gap, Yang and his colleagues in China have broadened the emphasis of uncertainty. They proposed a conceptual framework for uncertainty stress, and defined uncertainty stress as anxiety in facing ambiguous situations and problematic environments (Yang et al., 2007; Peng et al., 2019). Uncertainty is a common phenomenon in people’s everyday lives. It not only includes future uncertainty but also current uncertainty (Peng et al., 2019). Yang et al. (2019) considered the effects of rapid socio-economic transition, increased job competition, immature social values, and feelings of social anomie as sources of uncertainty stress. Drawing on theories of stress diathesis and theories of control and defense mechanisms (Mirowsky and Ross, 1990), they have shown that uncertainty stress and certainty stress like life stress or study stress, although related, are distinct components of stress (Wu et al., 2016, 2018; Yang et al., 2017b, 2018; Peng et al., 2019). Yang et al. (2017a) found that the prevalence of severe uncertainty stress among Chinese college students was 19.6%, while severe life stress prevalence was 8.6%. Some studies have provided empirical evidence that uncertainty stress, in particular, was associated with a range of negative health outcomes, including self-reported short- and long-term illness, alcohol abuse, unintentional injuries, deliberate self-harm, and suicidal ideation among Chinese college students (Wu et al., 2016, 2018; Yang et al., 2017b, 2018; Peng et al., 2019). However, no studies hitherto have examined uncertainty stress, in concert with other types of stress, and their relative impact upon mental health.

While a plethora of studies on uncertainty stress and its impact on psychological maladies with clinical patients have been conducted in western countries, these theoretical frameworks might not be directly transferable to the Chinese general population because of the complex Chinese social structure and the focus on traditional culture. The paucity of empirical research examining the association between uncertainty stress and mental disorders was justification for this study. The purpose of this study was to distinguish the effects of different stressors on mental health problems and explore the important role of uncertainty stress on the development of mental disorders. Given that the studies using a population-based sampling method with nationwide representativeness were limited globally, the present nationwide survey could add stronger empirical evidence to the association of stress especially uncertainty stress and mental health problems. Building upon the literature mentioned above, two hypotheses were developed for this study: (1) uncertainty stress would be negatively associated with the mental health of college students (Greco and Roger, 2003; Lee et al., 2004; Holland, 2016) and (2) uncertainty stress would be a stronger correlate to mental disorder than life stress or study stress (Wu et al., 2016, 2018; Yang et al., 2017b, 2018).

## Materials and Methods

### Data Source

The present study used the extended database from the 2013 Global Health Professions Student Survey (GHPSS) on Tobacco Control in China. The Chinese GHPSS Extended version included additional health-related information on perceived stress and mental health compared to the original version (Warren et al., 2008).

A multistage sampling strategy was utilized to recruit participants by geographic areas, university, and specific classes within each university. In stage 1, 50 universities were finally selected based on regional diversity, existing research collaboration with the principal investigator, and the site investigators’ willingness to participate. In stage 2, the sampling strategy involved the selection of levels within each university. All levels that had health professional courses were selected in each university. In stage 3, one-third of health-focused classes were randomly selected from each level. In stage 4, all students enrolled in those classes were recruited as study participants. The survey was conducted in class, where the principal investigators of each university explained the study to the students and obtained their informed consent before administering the survey in the classrooms. The questionnaires were collected by trained researchers after the participants completed them in class. Very few students refused to participate; thus, the response rate was very high. A detailed description on sampling, recruitment, and procedure can be found in Yang et al. (2015). The ethics committee at the Medical Center of Zhejiang University approved the study protocol (reference number: PJ2014035).

## Measures

### Dependent Variable

#### Mental Health Status

The mental health status of participants was measured by the 12-item Chinese Health Questionnaire (CHQ). The CHQ was developed in Taiwan and was derived from the General Health Questionnaire (GHQ) (Cheng and Williams, 1986; Chong and Wilkinson, 1989). Similar to the widely used GHQ, the CHQ was designed to screen for mental disorders in community settings (Cheng and Williams, 1986; Chong and Wilkinson, 1989; Goldberg and Williams, 1991). The CHQ version utilized in the current study was the Mandarin version, which was revised and adapted by Yang et al. (2003) according to Chinese mainland culture. It also has an acceptable reliability and validity and has been widely used to assess mental health status in community and primary care settings across China (Qiu et al., 2006; Ma et al., 2007; He et al., 2008). The CHQ included questions concerning somatic disorders, such as headaches, palpitations, sleeplessness, and other questions related to concerns about interpersonal relationships, confidence, and so on; for example, “Have you recently been suffering from shaking or numbness of your limbs?” and “Have you recently been losing confidence in yourself?” The internal reliability of the CHQ in this sample, as measured by Cronbach’s α coefficient, was 0.70, which suggested an acceptable level of reliability. Items in this questionnaire were rated on a four-point scale. The responses of “not at all” and “same as usual” were recoded as 0, and “rather more than usual” and “much more than usual” was recoded as 1. The summed score was calculated to measure the severity of mental disorders. Following previous practice, a cut-off score of 3 or more signified a mental disorder. This cut-off score was classified by the performance of the receiver operating characteristic curve using final clinical diagnosis as the gold standard in determining a mental disorder. It demonstrated both good sensitivity and specificity (Chong and Wilkinson, 1989; Yang et al., 2003).

### Independent Variables

#### Sociodemographic Characteristics

Information on several sociodemographic characteristics was collected including age, gender, ethnicity, grade, major, parental occupation, monthly expenditure, and family residence location.

#### University Contextual Factors

University type was determined using the China university ranking system (high, middle, and low level) as established by the National Ministry of Education. University location was geographically divided into west, middle, and east region of China.

#### Perceived Stress

Three types of stress were measured by the Student Daily Stress Questionnaire (SDSQ) developed by Yang et al. (2007). The life stress consisted of five items and included questions on financial, health, interpersonal relationships, family, and other issues of daily life. Study stress was assessed by three items that included heavy academic responsibilities, uninteresting professional curricula, and study environment. The uncertainty stress questionnaire consisted of four items, including current status uncertainty, social change uncertainty, goal uncertainty, and social value uncertainty (Yang et al., 2007; Wu et al., 2016). Items were rated on a five-point scale ranging from 0 (no stress) to 4 (excessive stress). Item scores were summed to attain a total stress score. The higher the total score, the greater is the perceived level of stress. Consistent with prior practice, scores exceeding two on each item indicated “high stress” (Yang et al., 2007, 2012, 2017a; Wu et al., 2016). The Cronbach’s α coefficient was 0.80 for uncertainty stress, 0.74 for life stress, and 0.61 for study stress in this study. These values suggested that the questionnaires had an acceptable reliability.

#### Data Analysis

All survey data were entered into a database using Microsoft Excel and then imported into SPSS (version 22.0) for statistical analysis. Descriptive statistics were calculated to determine the prevalence of mental disorders across different sociodemographic characteristics and stressors. All variables were categorical. We conducted unconditional logistic regression analysis to test hypotheses about associations between the independent variables and dependent variable. Both unadjusted and adjusted models were established to examine and confirm these associations. A nested hierarchical multilevel modeling technique was utilized, which has substantial advantages over a single-level regression procedure. Ecologic and atomistic fallacies were precluded by modeling random variation at both individual and university levels, thus providing the capacity to differentiate individual and contextual effects upon mental disorder outcomes (Wang et al., 2008).

Several models were built in this study. We started with the Null Model a two-level (individual and university) model with random intercepts, which only included a constant in accounting for variation in mental disorder across 50 universities. From this base model, we entered all individual- and university-level variables as fixed main effects, which were significantly associated with mental disorder from the unadjusted models, to construct four further models for evaluating the impact of variables on mental disorders. First, we constructed a model, which included study stress and life stress (Model 1). The second model included life stress and uncertainty stress. Model 3 added study stress and uncertainty stress. The final model (Model 4) included three types of stress. Any confounding effects of significant factors on mental disorder were all controlled in Models 1–4. The models, thus, enabled us to examine the relative impacts of stress as predictors of mental disorder.

## Results

A total of 11,954 college students from 50 different universities across China completed the survey with a valid response rate of 97.5%. Of these, 15.8% were younger than 20 years old, 63.6% were from 20 to 22 years old, and 20.5% were 23 years old or more. Freshmen and sophomores accounted for 41.4%. Of the study sample, 35.6% were male students and 64.4% were female students. The majority of participants (93.3%) were of Han Chinese ethnicity and around 80% of the students’ parental occupations were in the categories of operations or commercial work. Nearly four-fifths of the students paid 1,000–1,999 RMB per month. More than half of the participating students were studying at universities located in the eastern region of China, and 35.9% of the participant students studied at universities ranked as high level (see Table 1).

**TABLE 1:** Sociodemographic characteristics and stress status of sample and mental disorder prevalence (*n* = 11,954).

		**% of**	**Prevalence**	
**Group**	***N***	**sample**	**(95% CI)**	**Unadjusted OR**
**Sociodemographic factors**				
**Age (years)**				
<20	1,894	15.8	20.7 (18.9–22.6)	1.00
20–	2,392	20.0	20.9 (19.2–22.5)	1.01 (0.87–1.17)
21–	2,762	23.1	23.3 (21.7–24.8)	1.16 (1.01–1.33)*
22–	2,450	20.5	22.3 (20.6–24.0)	1.10 (0.95–1.27)
23–	2,456	20.5	26.1 (24.4–27.8)	1.35 (1.17–1.56)**
**Gender**				
Male	4,253	35.6	19.6 (18.4–20.8)	1.00
Female	7,701	64.4	24.5 (23.5–25.5)	1.33 (1.21–1.46)**
**Grade**				
1–2	4,945	41.4	21.3 (20.2–22.5)	1.00
3–4	6,717	56.2	23.6 (22.5–24.6)	1.14 (1.04–1.24)**
5–	292	2.4	29.1 (23.9–34.4)	1.51 (1.17–1.97)**
**Ethnicity**				
Han	11,148	93.3	22.9 (22.1–23.6)	1.00
Minority	806	6.7	21.5 (18.6–24.4)	0.93 (0.78–1.10)
**Major**				
Medical	10,507	87.9	23.2 (22.4–24.0)	1.22 (1.06–1.40)**
Others	1,447	12.1	19.8 (17.8–21.9)	1.00
**Monthly expenditure**				
<1,000	1,299	10.9	22.6 (20.3–24.8)	1.00
1,000–1499	6,165	51.6	24.3 (23.2–25.3)	1.10 (0.95–1.27)
1,500–1,999	3,506	29.3	20.5 (19.1–21.9)	0.89 (0.76–1.03)
2,000 and more	984	8.2	21.5 (18.9–24.1)	0.94 (0.77–1.15)
**Father’ s occupation**				
Operation and commercial work	9,457	79.1	22.9 (22.0–23.7)	1.00
Staff and administration	1,741	14.6	21.9 (20.0–23.9)	0.95 (0.84–1.07)
Professionals	756	6.3	23.1 (20.1–26.2)	1.02 (0.85–1.21)
**Mother’s occupation**				
Operation and commercial work	9,600	80.3	23.1 (22.2–23.9)	1.00
Staff and administration	1,548	12.9	21.3 (19.2–23.4)	0.90 (0.79–1.03)
Professionals	806	6.7	22.0 (19.1–24.9)	0.94 (0.79–1.12)
**University contextual factors**				
**University location**				
East	6,615	55.3	21.8 (20.8–22.9)	1.00
Middle	3,524	29.5	23.9 (22.5–25.3)	1.12 (1.02–1.24)*
West	1,815	15.2	23.8 (21.8–25.8)	1.12 (0.99–1.26)
**Type of university**				
High level	4,295	35.9	21.1 (19.9–22.3)	1.00
Middle and low level	7,659	64.1	23.7 (22.8–24.7)	1.17 (1.07–1.28)**
**Psychological factors**				
**Study stress**				
Low score	9,636	80.6	20.0 (19.2–20.8)	1.00
High score	2,318	19.4	34.2 (32.3–36.2)	2.08 (1.88–2.30)**
**Life stress**				
Low score	10,960	91.2	21.6 (20.9–22.4)	1.00
High score	1,048	8.8	34.5 (31.6–37.4)	1.91 (1.67–2.19)**
**Uncertainty stress**				
Low score	9,614	80.4	19.2 (18.4–20.0)	1.00
High score	2,340	19.6	37.6 (35.6–39.5)	2.54 (2.30–2.80)**

*p < 0.05; **p < 0.001.

**FIGURE 1:**
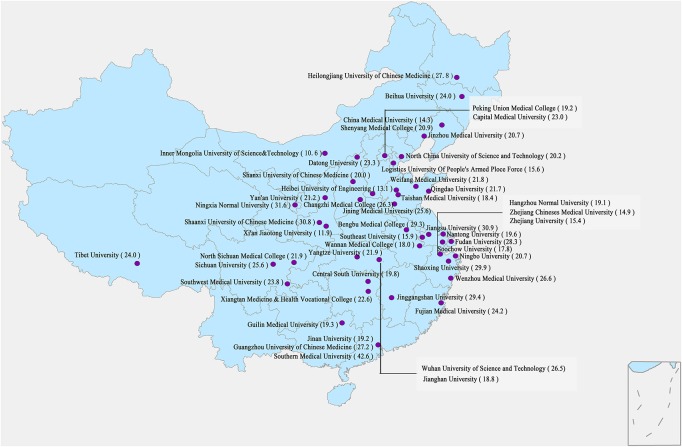
Geographical distribution and the prevalence of mental disorder of the 50 universities across China.

The total mental disorder prevalence among this sample of college students was 22.8% (95% CI: 22.0–23.5%). Table 1 shows mental disorder prevalence across sociodemographic characteristics. Older students had a higher prevalence of mental disorders than did younger students. The prevalence of mental disorders among female students (24.5%) was higher than that of male students (19.6%). Medical students had a mental disorder prevalence of 23.2%, which was higher than that of non-medical students (19.8%). Figure 1 displays the geographical distribution and prevalence of mental disorders at the 50 participating universities. The 25th, 50th, and 75th quartiles of prevalence of mental disorder for universities were 19.0, 21.8, and 26.4%, respectively. The unadjusted model indicated that age, gender, grade, major, and university location and type were all associated with mental disorders among students. Ethnicity, monthly expenditure, and parental occupation were not associated with mental disorders. According to the unadjusted logistical models, students who were 23 or more years old were most likely to experience mental disorders. There was a gender difference in the prevalence of mental disorders with female students exhibiting a higher odds ratio (OR: 1.33) than that of male students. The odds of a mental disorder among medical students were 1.22 times higher than that of non-medical students. The likelihood of a mental disorder among students who studied in the middle region of China was 1.12 times higher than that of those who studied in the eastern region of China. Students who studied at the high-level universities were 1.17 times more likely to experience mental disorders than those students who studied at middle- and lower-level universities.

As to psychological factors, the unadjusted models demonstrated that study stress, life stress, and uncertainty stress were all positively associated with mental disorders. Multilevel logistic regression Model 1 in Table 2 showed that both study stress and life stress were associated with mental disorder. However, once uncertainty stress was taken into account in Model 2 and Model 3, life stress and study stress had limited utility for predicting mental disorders. The odds ratio of uncertainty stress was higher than that of life stress and study stress. The adjusted full model (Model 4) revealed that students who perceived higher study stress and uncertainty stress were 1.43 and 2.55 times more likely to have a mental disorder than those with lower stress scores, but life stress was not significantly associated with mental disorders. The fixed parameters in models 2–4, which included uncertainty stress, were decreased in comparison with those in Model 1. This indicated that uncertainty stress could explain the higher variation for mental health problems and further disclosed that it was an important risk factor for mental disorders.

**TABLE 2:** Findings of multilevel analysis on mental disorders by different types of stress.^#^

**Group**	**Null model**	**Model 1**	**Model 2**	**Model 3**	**Model 4**
**Study stress**					
Low score		1.00		1.00	1.00
High score		1.91 (1.61–2.26)**		1.46 (1.17–1.82)**	1.43 (1.17–1.74)**
**Life stress**					
Low score		1.00	1.00		1.00
High score		1.61 (1.12–2.23)**	1.34 (0.83–2.15)		1.22 (0.78–1.92)
**Uncertainty stress**					
Low score			1.00	1.00	1.00
High score			2.83 (2.17–3.64)**	2.20 (2.06–3.37)**	2.55 (1.94–3.35)**
Fixed parameters	4.2075**	3.947**	3.6162**	3.6016**	3.7858**
Random parameters between universities	0.1758**	0.1360**	0.1462**	0.1443**	0.1450**

**p < 0.01. ^#^Model 1 included study stress and life stress. Model 2 contained life stress and uncertainty stress. Model 3 included study stress and uncertainty stress. The full model (Model 4) included three types of stress. The sociodemographic and contextual variables listed in Table 1 were all controlled from Model 1 to Model 4.

## Discussion

Based on the results of this study, more than one-fifth of the college students reported mental disorders, which revealed that mental health is an urgent and salient issue that requires immediate attention and intervention. We found a number of sociodemographic and college-related variables that had statistically significant associations with overall mental disorder prevalence. Based on our literature review, this is the first multi-university study to demonstrate these relationships in China. Students who were 23 years old and more had a greater likelihood of mental disorder. The higher the grade the students attended, the higher risk for mental disorder. This might be related with the increased stress in the later period of university and the need for students to begin considering employment after graduation. Consistent with previous studies, female students in this study experienced a higher rate of mental disorders than male students (Auerbach et al., 2018; Bruffaerts et al., 2018). Medical students had a greater likelihood of experiencing a mental disorder than non-medical students. This is probably due to the heavy academic burden associated with medical school. Students who studied at universities located in the middle region of China had a higher risk for mental disorders than did students who studied at eastern universities. One possible explanation for this might be regional inequities in educational resources and economic development. Studying under these less-than-ideal conditions might impact mental health. There was also a significant relationship between university type and mental disorders. This might reflect the fact that students attending high-level universities have a better learning environment with more resources and services to support their mental health. Identifying high-risk students for mental disorders provides critical information for preventative-intervention efforts.

This study adds to the literature by demonstrating that uncertainty stress is uniquely associated with mental disorders in a college-age population. In this undergraduate sample, uncertainty stress shows a stronger correlation with mental disorders than study stress or life stress. It expands the present body of knowledge concerning the relationship between stress and mental disorders. The findings from this study were congruent with those from a previous research study on uncertainty stress with self-reported physical illness (Yang et al., 2017b). The negative effect of uncertainty stress on perceived mental health was supported and confirmed by the empirical data of this study (Kang, 2005).

There are several plausible explanations for the findings related to uncertainty stress. Uncertainty occurs when an event or a situation causes ambiguity, inconsistency, or unpredictability (Mast, 1995). Uncertainty in illness theory asserts that uncertainty develops when a person is unable to attribute specific values to objects or events or is unable to predict outcomes because of a lack of sufficient cues (Mishel, 1988). Uncertainty stress can stem from lack of information or resources provided to college students about their current state and future endeavors. According to current studies, there are four forms of uncertainty stress: fate unpredictability, life goal uncertainty, social value uncertainty, and social change uncertainty (Wu et al., 2016; Yang et al., 2017a, b). One study underscored that socioeconomic inequalities were an antecedent of uncertainty stress (Yang et al., 2017a). In that study of college students, uncertainty stress was found to be related to one’s social standing, access to material resources, and levels of social support (Yang et al., 2017a). As predicted by the social exchange theory (Blau, 1964), students with a lower social status will have less economic power and will be challenged to cope with stressful situations (Yang et al., 2019). The life of a college student is uncertain. Not only is their uncertainty related to their academic progress but also their social lives and their future beyond college. Uncertainty regarding current status and life meaning, the complexity of achieving life goals, lack of information about future endeavors, and an inconsistent value orientation probably constitute potent and negative psychological stimuli for college students (Fortier et al., 2013).

China is in a critical stage of societal transformation, and the Chinese people are experiencing much more social change than in the past. Besides rapid economic development, the phenomenon of serious social anomie, widening gap between rich and poor, and the consolidation of social classes are all taking their toll (Yang et al., 2017b). Thus, uncertainty stress becomes a very real issue for much of society. One survey found that 43% of Chinese urban residents perceived moderate or severe stress caused by uncertainty (Yang et al., 2007). College students, as young adults, lack social experience and the mental maturity to prepare for entering this society with all its uncertainty. College students may face difficulty in envisioning their future due to the unpredictability of China’s future and the confusion of their own life goals. Students lack the cognitive maturity to deal with such societal issues, and this may lead to poor psychosocial adjustment. College students tend to feel uncertainty, insecurity, and self-doubt when confronted by the chaos of social values and the reality of rapid and deep social reform. Increased uncertainty may undermine an individual’s ability to sustain an acceptable quality of psychological and spiritual life (Sammarco and Konecny, 2008). Uncertainty stress may have a deleterious effect on mood state (Jiang and He, 2012). There is considerable evidence that intolerance of uncertainty creates a cognitive vulnerability that leads to worry and ultimately may result in generalized anxiety disorders (Ladouceur et al., 2000; McEvoy and Mahoney, 2011). Perceived uncertainty is not only directly correlated with anxiety and depression but also amplifies anxiety and depression, which, in turn, undermine the quality of life. It requires more avoidant and inactive emotional coping strategies than a student may possess (Gentes and Ruscio, 2011; Sharif et al., 2017). Uncertainty stress is often appraised as threatening or distressing and may create the perception that this is beyond one’s ability to cope (Johnson et al., 2006). Furthermore, when uncertainty is appraised as a danger rather than opportunity, it is associated with a pessimistic view of life (Kang, 2005). It makes sense that college students with higher degrees of uncertainty stress were more likely to experience mental disorder.

In comparison with study stress or general life stress, college students seem more sensitive and vulnerable to cognitive uncertainty. This study found that uncertainty stress had a more negative influence and adverse consequences on college students’ mental health than study or life stress. Uncertainty stress might be a unique precursor to mental disorders. One plausible explanation is that college students are intolerant of ambiguous states and regard uncertainty as a threatening and unacceptable presence. This may be influenced by traditional Chinese culture (McEvoy and Mahoney, 2011; Wu et al., 2016). Moreover, empirical evidence also suggests that uncertainty stress has a stronger correlation with problem alcohol use, deliberate self-harm, unintentional injuries, and suicidal intention than general life stress among college students (Wu et al., 2016, 2018; Yang et al., 2018; Peng et al., 2019). These negative behaviors could, in turn, further aggravate the deterioration of mental health. In addition, uncertainty stress damages mental well being by challenging one’s capacity to predict and plan in such a way as to be able to act efficaciously, and compared to more generic patterns of life stress, its coping requires more psychological resources because of the uncertainty trigger (Yang et al., 2019).

Based on the findings from this study, it would seem to be very important to empower college students to understand and embrace uncertainty from a cognitive perspective, and to cope with uncertainty stress correctly using stress management strategies. While students cannot eliminate all uncertainty, learning to live with uncertainty is important. Finding specific and clear life goals, enhancing skills to cope with uncertainty, supplying cognitive resources to make life adjustments or minimize internal conflict, and facilitating recognition of uncertainty might render uncertainty stress a less threatening presence (Mast, 1995). Learning how to manage stress through meditation, relaxation, regular exercise, and/or Yoga is important. Reframing cognitive thinking to guide the interpretation of an uncertain event or situation as less threatening may be an effective method for coping with uncertainty stress (Fortier et al., 2013). An intervention study for patients with chronic disease showed that an uncertainty management project can significantly improve the emotional status and mental health of clinical patients (Jiang and He, 2012). These findings suggest that uncertainty stress management and interventions for college students should be the focus in further work.

## Study Limitations

The cross-sectional nature of this study is an important limitation as it prevents establishment of a causal link between uncertainty stress and self-reported mental disorders. However, with such a large-scale sample, diversity of geographical spatial distribution, and the strength of some associations, the plausibility of cause and effect is increased. Future studies would benefit from a longitudinal examination of the correlation between these constructs. In addition, uncertainty stress intervention studies are needed to facilitate understanding how coping uncertainty stress influences students’ mental health in the next stage. Another limitation is that our participants were college students, and the majority of them were medical students. Thus, our results cannot be generalized to all college students or the wider Chinese population. Furthermore, different types of stress were measured using the self-developed scale. Although applying an instrument with an acceptable reliability, it is a preliminary exploration to assess uncertainty stress. More attention should be paid on the measurement of uncertainty stress. Finally, the mental health status of participants was measured by self-reported questionnaire rather than final clinical diagnosis from hospitals. However, the CHQ has an acceptable reliability and validity as a psychometric tool and has been widely used to assess mental health status in a community setting. For the population-based study, it is a relatively convenient and cost-effective method to screen for mental disorders. Enabled by appropriate funding, future research should utilize established clinical diagnostic methods to yield a more objective picture.

## Conclusion

The present study has provided evidence of the relationship between uncertainty stress and the mental health of Chinese college students. Compared with life and study stress, more attention should be given to uncertainty stress. Uncertainty stress has been overlooked as a key risk factor and robust predictor of mental disorders. It is important that college students be made aware of the harmful impact of uncertainty stress and be provided with strategies to manage and control such stress. The study identifies a new perspective for preventing mental health issues for policy makers and university administrators and helping to treat those with documented mental health problems. The findings from this study also strongly support the need for school-based intervention programs on improving coping skills for uncertainty stress among college students in China.

## Data Availability Statement

The datasets generated for this study are available on request to the corresponding author.

## Ethics Statement

The ethics committee of the Medical Center of Zhejiang University approved the study protocol, and informed consent was obtained from all individual participants.

## Author Contributions

TY designed the study. TY and DW coordinated the study and analyzed the data. DW and LY drafted the manuscript. TY and RC revised the manuscript. SP, WG, and SJ conducted the data collection.

## Conflict of Interest

The authors declare that the research was conducted in the absence of any commercial or financial relationships that could be construed as a potential conflict of interest.
